# Supplementary material for: A Microfluidic Platform for Actin‐Based Membrane Remodeling Reveals the Stabilizing Role of Branched Actin Networks on Lipid Microdomains
Source: Small Sci. 2025 Jul 22;5(10):2500210. doi: 10.1002/smsc.202500210 (PMC12499385; doi:10.1002/smsc.202500210)
Supplement: Supplementary file 1 — Supplementary Material [file SMSC-5-2500210-s001.pdf]

A microfluidic platform for actin-based membrane remodeling reveals the stabilizing role of branched actin networks on lipid microdomains.

Lixin Huang[1], Rogério Lopes Dos Santos[1], Sid Labdi[1], Guillaume Lamour[1], Olek Maciejak[1], Michel Malo[1], John Manzi[2], Martin Lenz[3], Jacques Fattaccioli[4,5] Clément Campillo[1,6]*

[1] Université Paris-Saclay, Univ Evry, CY Cergy Paris Université, CNRS, LAMBE, 91025 Evry-Courcouronnes, France;

[2]Institut Curie, Université PSL, Sorbonne Université, CNRS UMR168, Physique des Cellules et Cancer, 75005 Paris, FR.

[3]LPTMS, CNRS, Université Paris-Sud, Université Paris-Saclay, 91405 Orsay, France.

[4] CPCV, Département de Chimie, École Normale Supérieure, PSL University, Sorbonne Université, CNRS, 75005 Paris, France;

[5] Institut Pierre-Gilles de Gennes pour la Microfluidique, 75005 Paris, France.

[6] Institut Universitaire de France (IUF), 75231 Paris, France;

* Correspondence to: clement.campillo@univ-evry.fr

Supplementary materials

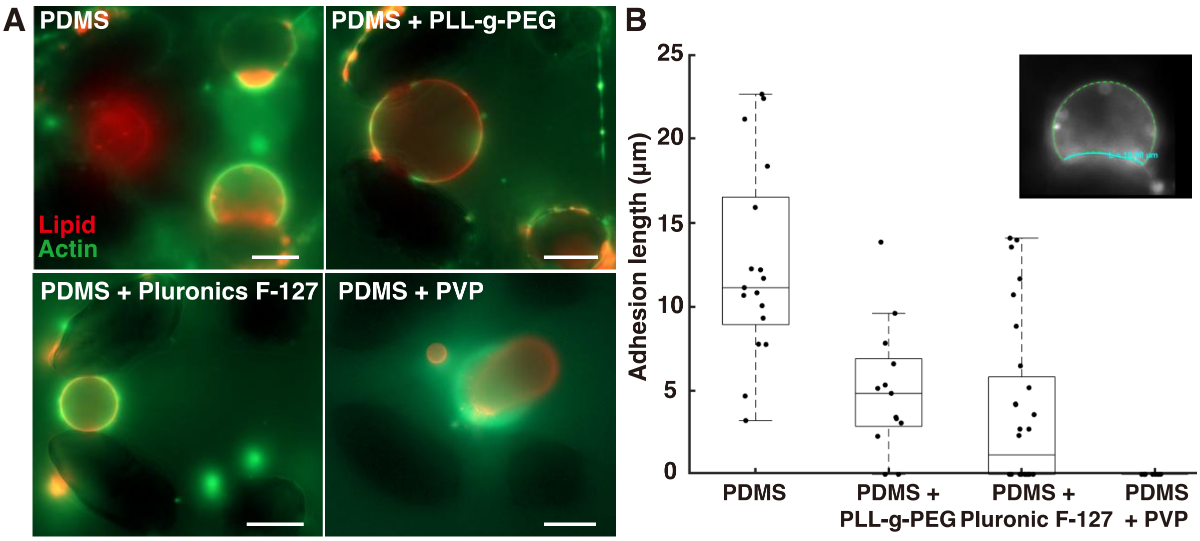

Figure S1. Impact of the microfluidic chamber coating A) Fluorescent images of GUVs in presence of actin and accessory proteins bare PDMS microtraps or PDMS coated by PEG-PLL, Pluronic F127 or PVP B) GUV adhesion length to the microtraps in the different conditions presented in A); insert: scheme of the GUV adhesion length measurement from fluorescent images.

Grafted substrate	Contact Angle (°)	Ref.	Molecular weight (kDa)	Supplier
PDMS only	110	[1–4]	/	Dow Corning
PLL-g-PEG	80-90	[1,3]	PLL = 20; PEG = 2	SuSoS
Pluronic F-127	26	[2]	12.6	Merck
PVP	12	[4]	10	Merck

Table S1. Assessing coating efficiency by contact angle measurements: published values on PDMS and coated-PDMS.

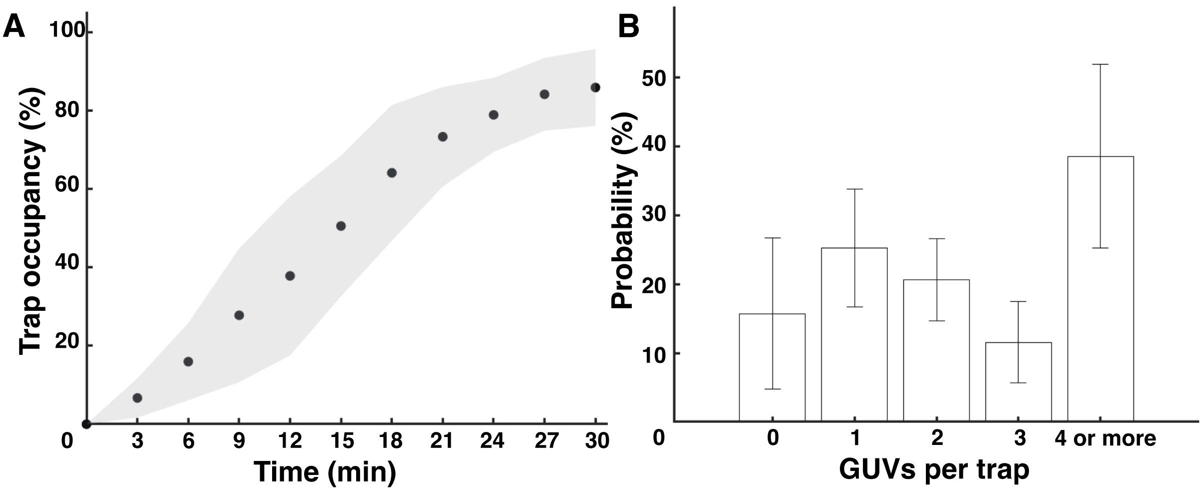

Figure S2. (C) Microtraps occupancy rate over time for a volumetric flow of 0.5 µl/min (mean ± standard deviation, N = 16 experiments); (D) Number of GUVs per microtrap (mean ± standard deviation, 1792 traps from 16 independent experiments).

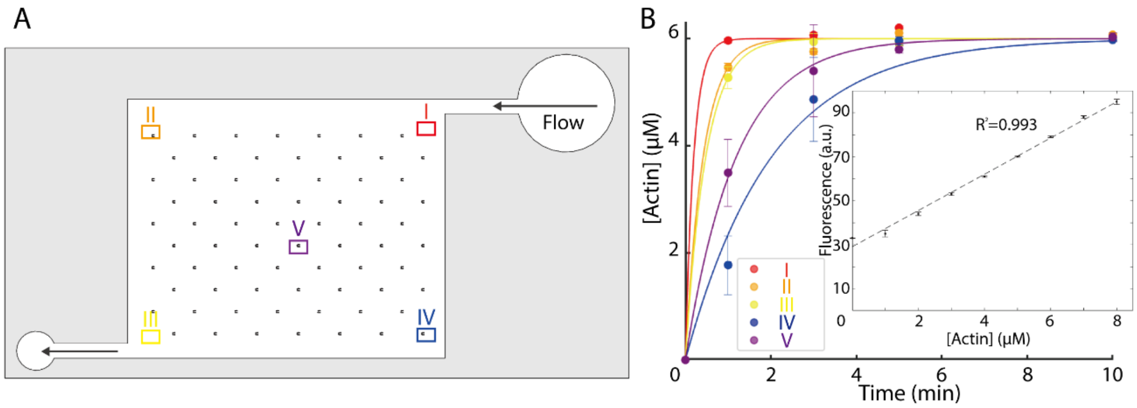

Figure S3: Quantitative analysis of actin concentration in the microfluidic chamber. (A) Scheme of the microfluidic chamber, with arrows indicating the direction of fluid flow and colored rectangles highlighting the Regions of Interest (ROIs) selected for quantifying actin concentrations; (B) Actin concentration vs time in ROIs (mean ± half of the standard deviation, N = 12 independent experiments, with sigmoidal fitting curves); Insert: Calibration of fluorescence intensity vs actin concentration in the microfluidic chamber (mean ± standard deviation, N = 12 experiments, with a linear fit: $R^2 = 0.993$).

First, we validate a linear relationship between actin fluorescent intensity and concentration (*Fig-S3B insert*). Then, we define five Regions Of Interest (ROIs) spanning the microfluidic chamber (*Fig-S3A*). As a static control, we add a 6 µM solution of fluorescent G-actin into the inlet reservoir (top right circle on *Fig-S3A)* without aspiration. In this case, after 15 minutes, no actin is observed in the microfluidic chamber, showing the stability of the pressure control. Then, we run the syringe pump at a flow rate of 0.5 µl/min for 10 minutes (*Fig-S3B*). The fluorescent intensity at the two top ROIs and the bottom left one (red, orange, and yellow rectangles in *Fig-S3A*) increases rapidly, and the G-actin concentration reaches the level measured in the inlet reservoir in 1 minute. In the ROIs at the center and the bottom-right (purple and blue rectangles in *Fig-S3A*), G-actin concentration reaches this value within 5 minutes.

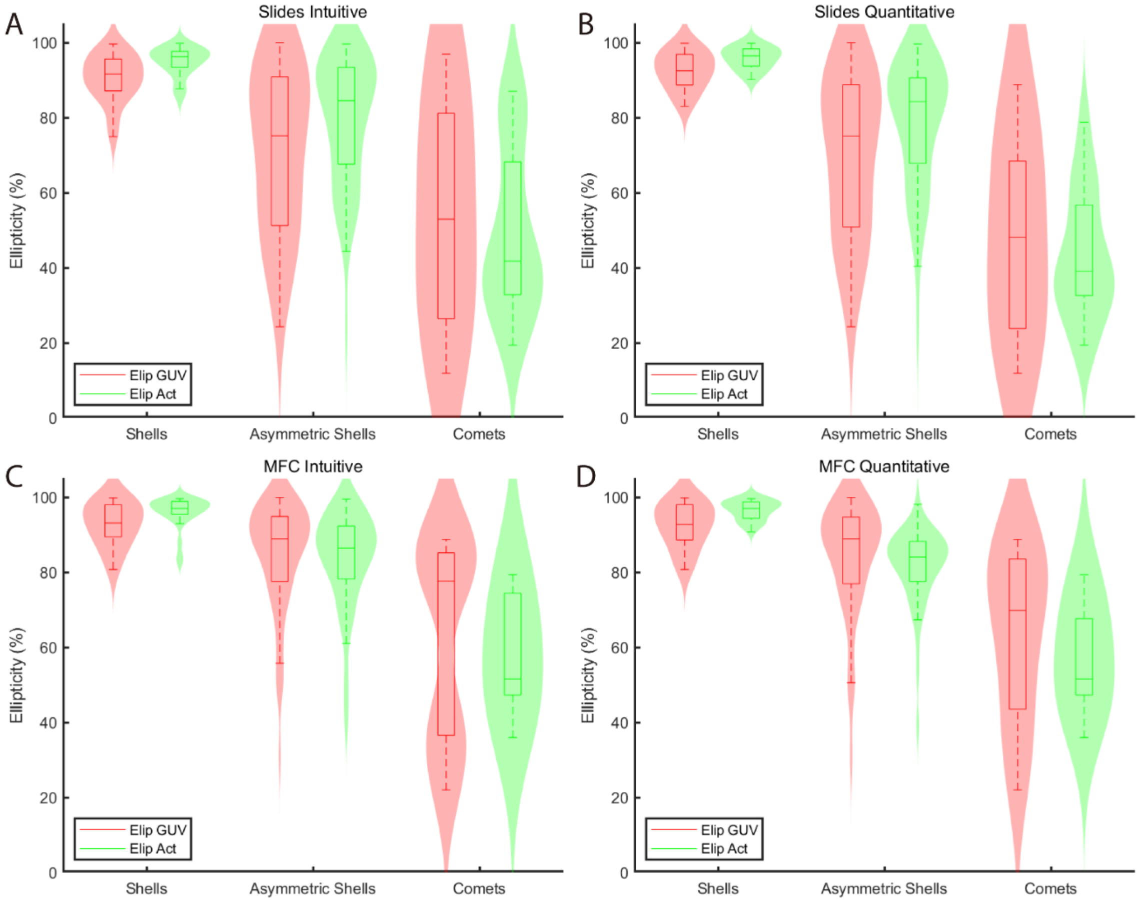

Figure S4. Ellipticity of GUVs and actin networks for three sorts of actin architecture. (A&C) Actin architectures are identified by eye for coverslips and MFC experiments; (B&D) Actin architectures are quantitatively classed according to their ellipticities for both coverslips and MFC experiments.

To classify the actin architectures, as a first step, the polymerized GUVs were intuitively distinguished by eyes and classed into 3 groups. Next, we measured the ellipticity for the remodeled lipid membranes and the actin network (*Fig-S2*). Seen the significant ellipticity differences among the 3 sorts of actin architecture, we then seek to explore a parametric fashion to separate them quantitatively. Symmetric actin shells are set to have GUV ellipticity higher than 0.8 and actin network ellipticity higher than 0.9; actin comets are set to have GUV ellipticity lower than 0.9, actin network ellipticity lower than 0.85, and actin thickness thicker than 7 µm; the rest is reckoned as asymmetric shells (*Fig-S2*)

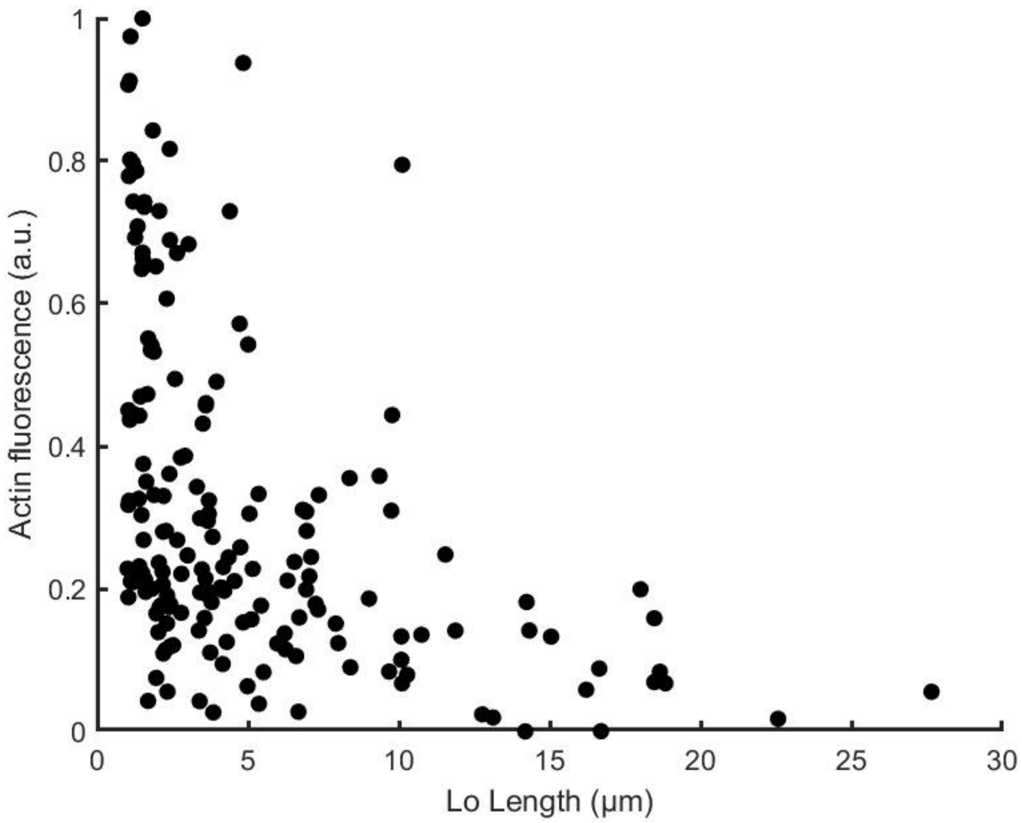

Figure S5. Actin fluorescence in Lo domains as a function of their length

Group(s)	Type of test	P-value	Comments
GUV10/0	Shapiro-Wilk	0.066	Normal
GUV60/0	Shapiro-Wilk	0.066	Normal
VCA10/0	Shapiro-Wilk	0.525	Normal
VCA60/0	Shapiro-Wilk	0.050	Normal
Actin10/0	Shapiro-Wilk	4e-08	Not normal
Actin60/0	Shapiro-Wilk	2e-07	Not normal
GUV10/0 vs GUV60/0	Fisher's F Variance	1	Same variance
VCA10/0 vs VCA60/0	Fisher's F Variance	3e-05	Diff. variance
GUV10/0 vs VCA10/0	Fisher's F Variance	5e-14	Diff. variance
GUV60/0 vs VCA60/0	Fisher's F Variance	1e-07	Diff. variance
GUV10/0 vs GUV60/0	Wilcoxon rank-sum test	1	n. s. diff.
VCA10/0 vs VCA60/0	Wilcoxon rank-sum test	9e-05	***
Actin10/0 vs Actin60/0	Wilcoxon rank-sum test	0.014	*
GUV10/0 vs VCA10/0	Mann-Whitney U test	9e-08	***
GUV10/0 vs Actin10/0	Mann-Whitney U test	1e-10	***
GUV60/0 vs VCA60/0	Mann-Whitney U test	0.034	*
GUV60/0 vs Actin60/0	Mann-Whitney U test	2e-10	***

Table S2. Statistical tests for Figure 6C.

Group(s)	Type of test	P-value	Comments
>12 domains	Shapiro-Wilk	0.308	Normal
4-10 domains	Shapiro-Wilk	0.039	Normal
2 domains	Shapiro-Wilk	0.098	Normal
>12 vs 4-10 domains	Fisher's F Variance	0.237	Same variance
>12 vs 2 domains	Fisher's F Variance	0.229	Same variance
4-10 vs 2 domains	Fisher's F Variance	0.789	Same variance
>12 vs 4-10 domains	Welch's t-tests	3e-10	***
>12 vs 2 domains	Welch's t-tests	2e-06	***
4-10 vs 2 domains	Welch's t-tests	0.062	n.s. diff.

Table S3. Statistical tests for Figure 7B.

Reference

[1] L. Rao, Y. Liu, H. Zhou, *J. Mater. Sci. Mater. Med.* **2022**, *33*, 66.

[2] T. Matsunaga, M. Hosokawa, A. Arakaki, T. Taguchi, T. Mori, T. Tanaka, H. Takeyama, *Anal. Chem.* **2008**, *80*, 5139.

[3] R. Kessler, **1999**, DOI 10.3929/ETHZ-A-004312236.

[4] S. Hemmilä, J. V. Cauich-Rodríguez, J. Kreutzer, P. Kallio, *Appl. Surf. Sci.* **2012**, *258*, 9864.
